# Overexpression of plastid lipid-associated protein in marine diatom enhances the xanthophyll synthesis and storage

**DOI:** 10.3389/fmicb.2023.1143017

**Published:** 2023-04-21

**Authors:** Er-Ying Jiang, Yong Fan, Nghi-Van Phung, Wan-Yue Xia, Guang-Rong Hu, Fu-Li Li

**Affiliations:** ^1^Shandong Provincial Key Laboratory of Synthetic Biology, Qingdao C1 Refinery Engineering Research Center, Qingdao Institute of Bioenergy and Bioprocess Technology, Chinese Academy of Sciences, Qingdao, China; ^2^University of Chinese Academy of Sciences, Beijing, China; ^3^Shandong Energy Institute, Qingdao, China; ^4^Qingdao New Energy Shandong Laboratory, Qingdao, China

**Keywords:** plastoglobules, fucoxanthin, eicosapentaenoic acid, fucoxanthin chlorophyll a/c-binding protein, xanthophyll cycles

## Abstract

Plastoglobules, which are lipoprotein structures surrounded by a single hydrophobic phospholipid membrane, are subcellular organelles in plant chromoplasts and chloroplasts. They contain neutral lipids, tocopherols, quinones, chlorophyll metabolites, carotenoids and their derivatives. Proteomic studies indicated that plastoglobules are involved in carotenoid metabolism and storage. In this study, one of the plastid lipid-associated proteins (PAP), the major protein in plastoglobules, was selected and overexpressed in *Phaeodactylum tricornutum*. The diameter of the plastoglobules in mutants was decreased by a mean of 19.2% versus the wild-type, while the fucoxanthin level was increased by a mean of 51.2%. All mutants exhibited morphological differences from the wild-type, including a prominent increase in the transverse diameter. Moreover, the unsaturated fatty acid levels were increased in different mutants, including an 18.9–59.3% increase in eicosapentaenoic acid content. Transcriptomic analysis revealed that PAP expression and the morphological changes altered xanthophyll synthesis and storage, which affected the assembly of the fucoxanthin chlorophyll a/c-binding protein and expression of antenna proteins as well as reduced the non-photochemical quenching activity of diatom cells. Therefore, metabolic regulation at the suborganelle level can be achieved by modulating PAP expression. These findings provide a subcellular structural site and target for synthetic biology to modify pigment and lipid metabolism in microalgae chassis cells.

## 1. Introduction

Diatoms, as a group of red phytoplankton in the ocean, contribute approximately 20% of the global primary productivity annually and play an essential role in the global carbon fixation and biogeochemical cycle (Field et al., 1998). Diatoms belong to an important branch of photosynthetic organisms. During evolution, prokaryotic cyanobacteria generated two major eukaryotic photosynthetic groups *via* endosymbiosis. One is the green branch, which includes Chlorophyta and land plants that evolved from Prochloron. The other is the red branch, which evolved from unicellular red algae that underwent endosymbiosis two or three times into various groups such as Cryptophyta, brown algae, diatoms, and dinoflagellates. Four membranes surround the chloroplasts of diatoms because of secondary endosymbiosis, differing from the double-membrane structure of chloroplasts in the common higher plants (Falkowski et al., 2004). The characteristic light-harvesting antenna of diatoms is fucoxanthin chlorophyll a/c-binding protein (FCP), which can capture blue and green light efficiently under low light conditions, thereby maintaining photosystem activity. This is the molecular basis for achieving efficient light energy capture (Wang et al., 2020). Meanwhile, diatoms can quench excessive excitation energy under high light conditions to prevent damage by reactive oxygen species, thereby giving diatoms strong light environment adaptation ability (Büchel, 2014).

As a marine model diatom, *Phaeodactylum tricornutum* has obvious advantages because of its intracellular content of biologically active substances, such as fucoxanthin and eicosapentaenoic acid (EPA). In recent years, fucoxanthin has been confirmed to be a safe and effective dietary supplement with various physiological activities, such as anti-inflammatory, anti-tumor, anti-obesity, and anti-diabetes activities (Maeda et al., 2007; Rokkaku et al., 2013; Martin, 2015). As one of the ω-3 series of polyunsaturated fatty acids (PUFAs), EPA prevents coronary cardiovascular disease and hypertriglyceridaemia and reduces the risk of arteriosclerotic inflammation and various neoplasias (Lorente-Cebrian et al., 2015; Souza and Norling, 2016). The use of biotechnology to improve the levels of fucoxanthin and EPA in *P. tricornutum* has great economic value. In this process, the synthetic pathways of these natural products need to be clarified and optimized.

Plastoglobules (PGs) were originally discovered in the chloroplast lamellae of *Euglena*, and they were characterized as dense osmiophilic globular structures (Wolken and Palade, 1952). Initial studies on PGs revealed that they are formed during the budding of the thylakoid membrane in chloroplasts. PGs attach to the thylakoid membrane and serve as an important site of chloroplast lipid storage, and they possess a single-layered membrane embedded with various proteins (Austin et al., 2006). These structures have spread widely in plastids of different types and developmental times, including chromoplasts and leucoplasts. PGs are relatively large in senescent cells, ranging 0.3–5.0 μm in size (Kaup et al., 2002). Differences in the size and morphology of PGs might serve as a cytological indicator of the growth status and stress tolerance. Among the PGs in the chromoplasts of ripening bell pepper fruits, capsanthin is the most abundant carotenoid, followed by violaxanthin, β-carotene, and capsorubin (Deruere et al., 1994b).

PGs are composed of lipoproteins coated by a monolayer of hydrophobic phospholipid membranes. They have four major components, namely neutral lipids, tocopherols and quinones, carotenoids and their derivatives, and chlorophyll catabolite (van Wijk and Kessler, 2017). According to proteomic analysis, the most abundant proteins in chloroplast PGs are specific members of the plastid lipid-associated protein (PAP)/fibrillin family (pfam04755) and members of the activity of BC1 complex kinase family, which represent approximately 53 and 19% of the PG protein mass, respectively (Singh and McNellis, 2011; Lundquist et al., 2012a). PAP/fibrillin was first isolated from the PGs of sweet pepper in 1994 (Deruere et al., 1994b). Thus far, the PAP/fibrillin family has been recognized as highly conserved, and it is divided into 12 subfamilies in higher plants. The results of subcellular localization experiments revealed that PAP/fibrillin proteins were located in various subcellular organelles of chloroplasts, such as the chloroplast stroma, thylakoid membranes, and PGs (Laizet et al., 2004; Singh and McNellis, 2011; Kim et al., 2018). *Arabidopsis* possesses 14 PAP/fibrillin proteins (termed FBNs in *Arabidopsis* in the following context), 7 of which (FBN1a, FBN1b, FBN2, FBN4, FBN7a, FBN7b, and FBN8) are considered as PG core proteins. The other FBNs are mainly localized in the thylakoid membranes (FBN3a/3b, FBN6, and FBN9) or chloroplast stroma (FBN5) (Lundquist et al., 2012b; Kim E. H. et al., 2015). The functions of FBN1, FBN2, and FBN4, which are localized in PGs, have been validated (Singh et al., 2010; Singh and McNellis, 2011). For example, overexpression of *FBN1a* in tobacco results in an increased number and larger size of PGs, along with increased tolerance to light stress, indicating that this gene functions in responses to biotic stresses (Rey et al., 2000). Knockdown of *FBN1* and *FBN2* in *Arabidopsis* resulted in a similar phenotype as the jasmonate-deficient mutant. Thus, FBN1a, FBN1b, and FBN2 help to recruit jasmonate biosynthetic enzymes to PGs (Youssef et al., 2010). In addition, the PAP/fibrillin family exhibits sequence conservation in the N- and C-terminal regions, including a lipocalin (−like) signature. Based on the presence of a lipocalin (−like) signature in PAP/fibrillin members, they are speculated to contribute to PG function through the binding and exchange of prenyl lipid intermediates. The identified functions of PG core proteins mainly involve the regulation of isoprenoid metabolism and remobilization of thylakoid fatty acids (van Wijk and Kessler, 2017).

Through the proteomic analysis of chromoplast PGs in ripe red peppers, many enzymes related to bicyclic carotenoid biosynthesis have been identified, such as ξ-carotene desaturase (ZDS), lycopene β-cyclase (LCY-β), and β-carotene β-hydroxylase. This suggests that chromoplast PGs have enzymatic functions in carotenoid biosynthesis (Ytterberg et al., 2006). The halotolerant green alga *Dunaliella bardawil* also has PGs with abundant β-carotene content. Analysis of its proteome revealed that it resembles eyespots in *Chlamydomonas reinhardtii* and the chloroplast PGs in *Arabidopsis*. Meanwhile, several enzymes that participate in β-carotene synthesis were identified, including one phytoene synthase gene, two phytoene desaturase genes, two LCY genes, four ZDS genes, and three carotene isomerase genes. Thus, the authors inferred that the abundant β-carotene in *D. bardawil* is probably synthesized in PGs (Davidi et al., 2015).

With continuous research on the structure of PGs, chloroplast PGs are regarded as highly specialized thylakoid microdomains that recruit and concentrate specific proteins and metabolites. In addition, chloroplast PGs play an active role in thylakoid formation, remodeling, and breakdown rather than merely serving a passive storage function as long believed (Brehelin et al., 2007; Besagni and Kessler, 2013). Peter K. Lundquist suggested that PGs are essentially microdomains within the thylakoid membrane, and they likely serve as a platform to recruit proteins and metabolites into spatial proximity, facilitating metabolic channeling or signal transduction to accomplish a series of metabolic functions (Lundquist et al., 2013). PGs comprise a type of microcompartment with integrated roles in plastid metabolism, developmental transitions, and environmental adaptation. Therefore, gene editing of PAP/fibrillin proteins could achieve rational regulation of cell growth metabolism in microalgae.

*P. tricornutum* is a unicellular organism with unique fucoxanthin synthesis and PUFA accumulation. Previous transmission electron microscopy (TEM) observation revealed that PGs are also present in the chloroplasts of *P. tricornutum*. Research on the pigment and functional protein composition of PGs provides an effective approach for further investigation into the synthesis of different bioactive components in diatoms, hence improving their accumulation.

In this study, a PAP/fibrillin protein in *P. tricornutum* was overexpressed, a large number of mutant strains were obtained, and their phenotypes were validated. We focused on the changes in fucoxanthin content. Concurrently, combined with the photosystem parameters and transcriptomics analysis, emphasis was also placed on the metabolic relationship between PGs and photosystem assembly. We dissected their synthetic regulatory mechanisms and investigated the effects of these mechanisms at the subcellular level.

## 2. Materials and methods

### 2.1. Microalgal cultivation

The wild-type (WT) *P. tricornutum* strain was stored at the Microalgae Culture Center (MACC/B228) of Ocean University of China. During autotrophic cultivation, algal cells were cultured in a modified f/2 medium with increasing sodium nitrate concentrations (1 g·L^−1^). The growth rate of *P. tricornutum* in mixotrophic cultivation can increase significantly. Therefore, 10 g·L^−1^ glycerol was added as the carbon source, and 2 g·L^−1^ tryptone was used as the nitrogen source. All strains were incubated in cell culture flasks and placed in a shaker at 24°C with a rotational speed of 160 r·min^−1^. The light intensity was 80 μmol photons m^−2^·s^−1^.

### 2.2. Selection of genes and plasmid construction

All *PAP* homologs in *P. tricornutum* and *A. thaliana* were jointly subjected to the phylogenetic tree analysis. Evolutionary analyses were conducted in MEGA11. The evolutionary history was inferred using the maximum likelihood method and Whelan and Goldman + Frequence model. The bootstrap consensus tree inferred from 1,000 replicates represents the evolutionary history of the taxa analyzed. Branches corresponding to partitions reproduced in fewer than 50% of bootstrap replicates were collapsed. The percentage of replicate trees in which the associated taxa clustered together in the bootstrap test 1,000 replicates are presented next to the branches (Tamura et al., 2021). Motifs were analyzed using the online tool MEME (Bailey and Elkan, 1994). Protein domains were predicted using SMART (Letunic et al., 2021). A highly homologous PAP gene (PHATRDRAFT_55153; XP_002184985.1) was finally selected. After sequence cloning, the gene was expressed using the plasmid pPha-T1.

### 2.3. Electroporation protocol

In total, 2 × 10^8^ cells of *P. tricornutum* during the exponential growth phase were harvested by centrifugation at 1500 × *g* for 10 min at 4°C. After 4–6 washes with 375 mM sterile ice-cold sorbitol, cells were resuspended in 100 μL of 375 mM sorbitol to a final density of 2 × 10^9^ cells·mL^−1^. Then, a suspension aliquot of 100 μL was mixed with 3–5 μg of DNA linear fragments and 4 μL (10 μg·μL^−1^) of salmon sperm DNA (denatured by boiling for 1 min), incubated on ice for 10 min, and then transferred into a 2-mm electroporation cuvette. Electroporation was performed using the following settings: 500 V, 400 Ω, and 25 μF (Hu and Pan, 2020). After electroporation, cells were immediately transferred to cell culture flasks containing 10–15 mL of fresh f/2 organic medium and recovered in low light (30 μmol photons m^−2^·s^−1^) overnight without shaking. Then, the cells were collected by centrifugation at 1500 × *g* for 10 min and resuspended in 600 μL of fresh f/2 organic medium, and 200 μL of this suspension were plated onto solid medium containing 75 μg·mL^−1^ bleomycin (Zeocin). Then, these plates were placed in an illumination incubator. After 5–7 weeks, the transformants were selected and transferred to liquid f/2 organic medium.

### 2.4. Determination of the photosynthetic system

The characteristics of the photosynthetic system of microalgae cells were measured as previously described (Ding et al., 2021). Each sample was collected, added to a black 96-well plate, and incubated for 10–15 min in the dark. Two experiments were performed in parallel. Then, the optimal/maximal quantum yield of PSII (Fv/Fm) and non-photochemical quenching (NPQ) were determined using an Imaging-PAM chlorophyll fluorometer (MAXI-Imaging-PAM, WALZ, Germany).

### 2.5. Rapid detection of fucoxanthin and chlorophyll *a* content in *Phaeodactylum tricornutum*

*P. tricornutum* cells were collected, and a portion was appropriately diluted. Then, 200 μL of each sample were added to a clear 96-well plate, and the optical density at 750 nm (OD_750_) was measured by a microplate reader to calculate the turbidity.

Meanwhile, the other portion of *P. tricornutum* cells was harvested by centrifugation at 4,000 × *g* for 5 min, washed once with distilled water, and centrifuged again. The cells were resuspended in ethanol (ethanol:microalgae = 1:1, *v/v*) for 15–20 min. After diluting, 200 μL of each sample were added to a clear 96-well plate, and OD_445_ and OD_663_ were measured using a microplate reader.

The content of fucoxanthin was calculated using the formula (Wang et al., 2018): fucoxanthin content (mg·L^−1^) = 6.39 × OD_445_–5.18 × OD_663_  + 0.312 × OD_750_–5.27.

*P. tricornutum* cells were collected and centrifuged at 4000 × *g* for 10 min at 4°C. The supernatant was discarded. An equal volume of 90% acetone was added, pipetted, and mixed. The samples were incubated at 4°C in the dark for 60 min. Then, the samples were centrifuged at 4,000 × *g* for 10 min at 4°C, and 200 μL of the supernatant were collected to determine OD_652_ and OD_655_. The Chl-a content was calculated using the following formula (Lichtenthaler, 1987): Chl-a (mg·L^−1^) = 16.72 × OD_665_–9.16 × OD_652_.

### 2.6. Analysis of the pigment composition and fucoxanthin concentration using HPLC

The pigment composition was measured using the Hitachi Primaide HPLC system (Hitachi, Tokyo, Japan) with a C18 reverse phase column (2.7-μm particle size, 100 × 4.6 mm). The mobile phase consisted of acetonitrile and water with a flow rate of 1 mL·min^−1^. In the gradient condition, the acetonitrile/water ratio was increased from 25:75 to 75:25 over 15 min, maintained for 3 min, and then decreased back to 25:75 over 2 min. The chromatogram was recorded at 445 nm. A fucoxanthin standard (ChromaDex, Irvine, CA, United States) was used to construct a standard curve in the 0.01–1.00 mg∙mL^−1^ range.

### 2.7. Determination of the EPA content by gas chromatography

To analyze the fatty acid profile, *P. tricornutum* cells were harvested by centrifugation at 4000 × *g* for 10 min, washed once with distilled water, and centrifuged again. The samples were placed in a freeze drier and lyophilized overnight, and their dry weight was determined. The lyophilized samples were mixed with chloroform and methanol (*v:v* = 2:1) and then shocked at 45°C for 3 h. Then, KCl (0.9%) was added to the samples, which were centrifuged at 4000 × *g* for 5 min. The bottom layer was transferred to a glass tube and weighed after drying with nitrogen gas. The lipid was added to n-hexane and sulfuric acid methanol (2%), and samples were placed in a baking oven for methyl esterification at 85°C for 2 h. Subsequently, the samples were removed and cooled on ice, and KCl (0.9%) was added. Then, samples were vortexed thoroughly and centrifuged at 4,000 × *g* for 5 min. Finally, the upper organic layer was taken for GC, which was performed using a GC system (7890A, Agilent Technologies, Inc., CA). INNOWAX (30 m × 320 μm × 0.25 μm) was selected as the chromatographic column. In the GC analysis program, an inlet temperature of 250°C and an injection volume of 1 μL were employed. The temperature program settings were 120°C for 5 min, linear ascension at 3.5°C·min^−1^ to 240°C, and a constant temperature for 10 min. N_2_ was utilized as the carrier gas with a speed of 28.5 mL·min^−1^ and split ratio of 10:1 (*v/v*).

### 2.8. Separation of lipid fraction

Polar and non-polar lipids in *P. tricornutum* were separated by using solid phase extraction column (Bond Elut SI, 500 mg/3 mL, Agilent, Santa Clare, CA). The 3 mL silica column was equilibrated by using 3 mL methanol and 9 mL chloroform. Then the lipids extracted from *P. tricornutum* were added into the column. Neutral lipids were eluted with 4.5 mL of chloroform:acetic acid (9:1, *v/v*). Glycolipids were eluted with 6 mL of acetone:methanol (19:1, *v/v*). And phospholipids were eluted with 6 mL of methanol (Damiani et al., 2010). The various lipid fractions were dried with nitrogen gas and added to n-hexane and sulfuric acid methanol (2%). The samples were taken for GC after methyl esterification at 85°C for 2 h as described above Materials and methods 2.7.

### 2.9. Transcriptomic analysis

On day 14, *P. tricornutum* cells were selected for transcriptomic analysis because the microalgae at this stage had relatively stable morphology and large variations in pigment content. Transcriptome sequencing and analysis were conducted by OE Biotech Co., Ltd. (Shanghai, China). Raw data (raw reads) were processed using Trimmomatic (Bolger et al., 2014). The reads containing ploy-N low-quality reads were removed to obtain clean reads. Then, the clean reads were mapped to the reference genome using hisat2 (Kim D. et al., 2015). The FPKM value of each gene was calculated using cufflinks, and the read counts of each gene were obtained by htseq-count (Trapnell et al., 2010).

### 2.10. Statistical analysis

All experiments were repeated thrice. Unless otherwise stated, all data are expressed as the mean ± standard deviation. The statistical significance of the values obtained from each experiment was evaluated *via* multiple *t*-tests using GraphPad Prism 8.0.2. Differences were considered significant at *p* < 0.05.

## 3. Results

### 3.1. Phylogenetic tree analysis of the PAP genes in *Phaeodactylum tricornutum*

**Figure 1:**
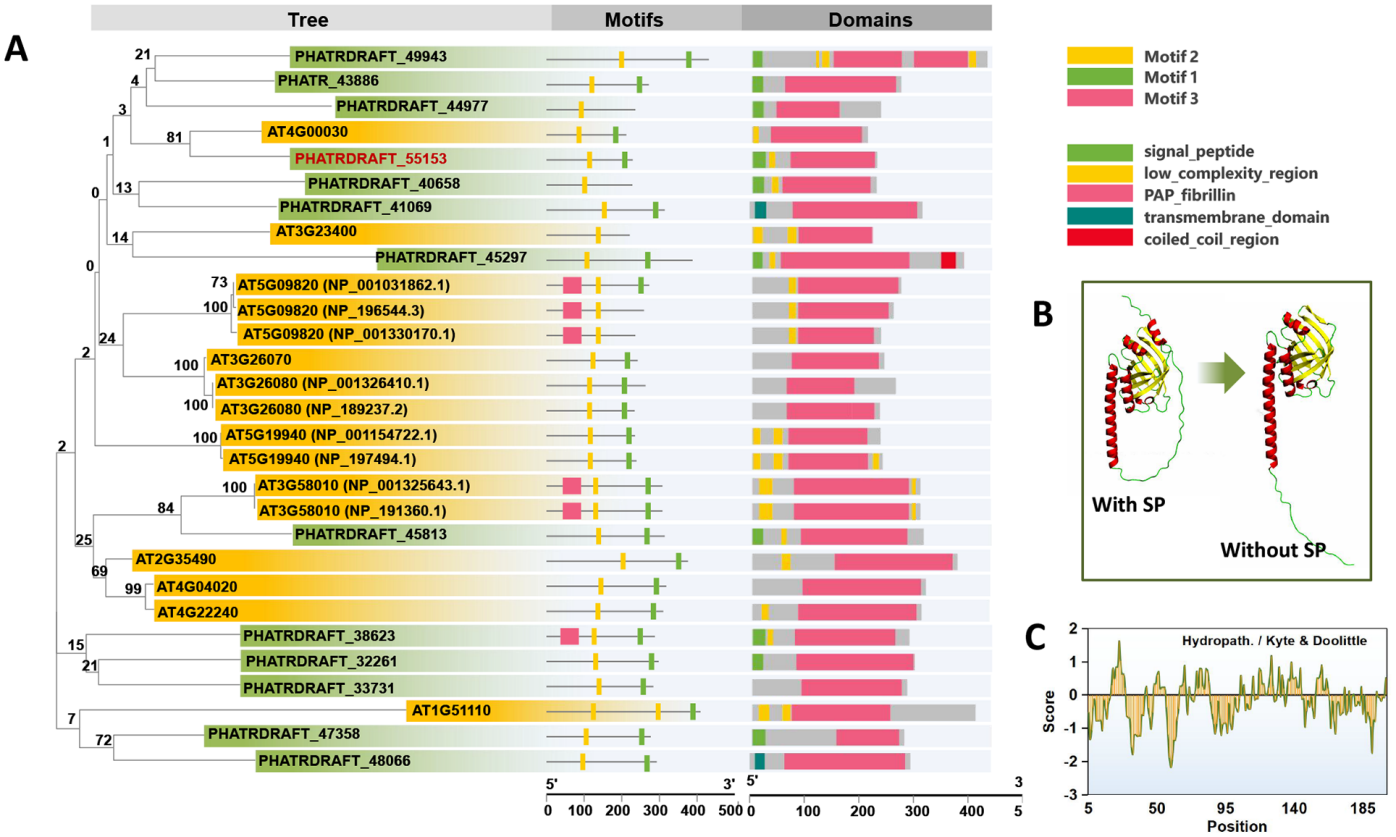
Sequence analysis of PAP proteins from *P. tricornutum*. **(A)** Phylogenetic tree analysis, motif analysis, and functional domain analysis of PAP/fibrillin proteins in *P. tricornutum* and *A. thaliana*. **(B,C)** Protein structure prediction and hydrophobicity analysis of PHATRDRAFT_55153. SP: signal peptide. All protein sequences are provided in Supplementary Data 1.

Thirteen homologous fragments of PAP proteins are found in *P. tricornutum*. These sequences were compared with the FBN proteins of *A. thaliana*. A phylogenetic tree was constructed using homology analysis (Figure 1A, Supplementary Data 1). Online analysis of the motifs using the MEME tool revealed that 2–3 fragments of conserved sequences are included in the protein sequence of the PAP/fibrillin family. The structures of all sequences were predicted and visualized using the SMART batch function in Tbtool software (Chen et al., 2020). We found that almost all of the PAP proteins in *P. tricornutum* have a signal peptide sequence or transmembrane domain. Among them, PHATRDRAFT_48066 and PHATRDRAFT_41069 have significant transmembrane domains. Excluding PHATRDRAFT_33731, all PAP proteins contained a chloroplast signal peptide. Meanwhile, PAP proteins in *A. thaliana* did not possess signal peptides and transmembrane domains. Previously, no PG protein was known or predicted to possess transmembrane domains, consistent with PGs being bound by a membrane lipid monolayer (van Wijk and Kessler, 2017). The presence of a signal peptide domain in *P. tricornutum* PGs might be related to their four-layered chloroplast membrane structure. Only a few PAP proteins of *P. tricornutum* are clustered into the same branch as the FBNs of *A. thaliana.* Among them, PHATRDRAFT_55153 and PHATRDRAFT_45813 clustered into one branch with AT4G00030 and AT3G58010, respectively, with bootstrap consensus values exceeding 80. Further combined with conserved sequence analysis, PHATRDRAFT_55153 has a more consistently conserved sequence similarity. Therefore, PHATRDRAFT_55153 was overexpressed in *P. tricornutum* to analyze the changes in PGs and their function in cell metabolism. The protein structure was predicted by Alphafold 2 (Jumper et al., 2021). The results illustrated that after the signal peptide removal, the protein had a long and straight N-terminus, and the C-terminus formed an eight-stranded antiparallel beta-barrel. Such structural and hydrophobic region characteristics might play special roles in the formation and maintenance of the monolayer structure (Figures 1B,C).

### 3.2. Overexpression of the PAP gene leads to morphological changes

**Figure 2:**
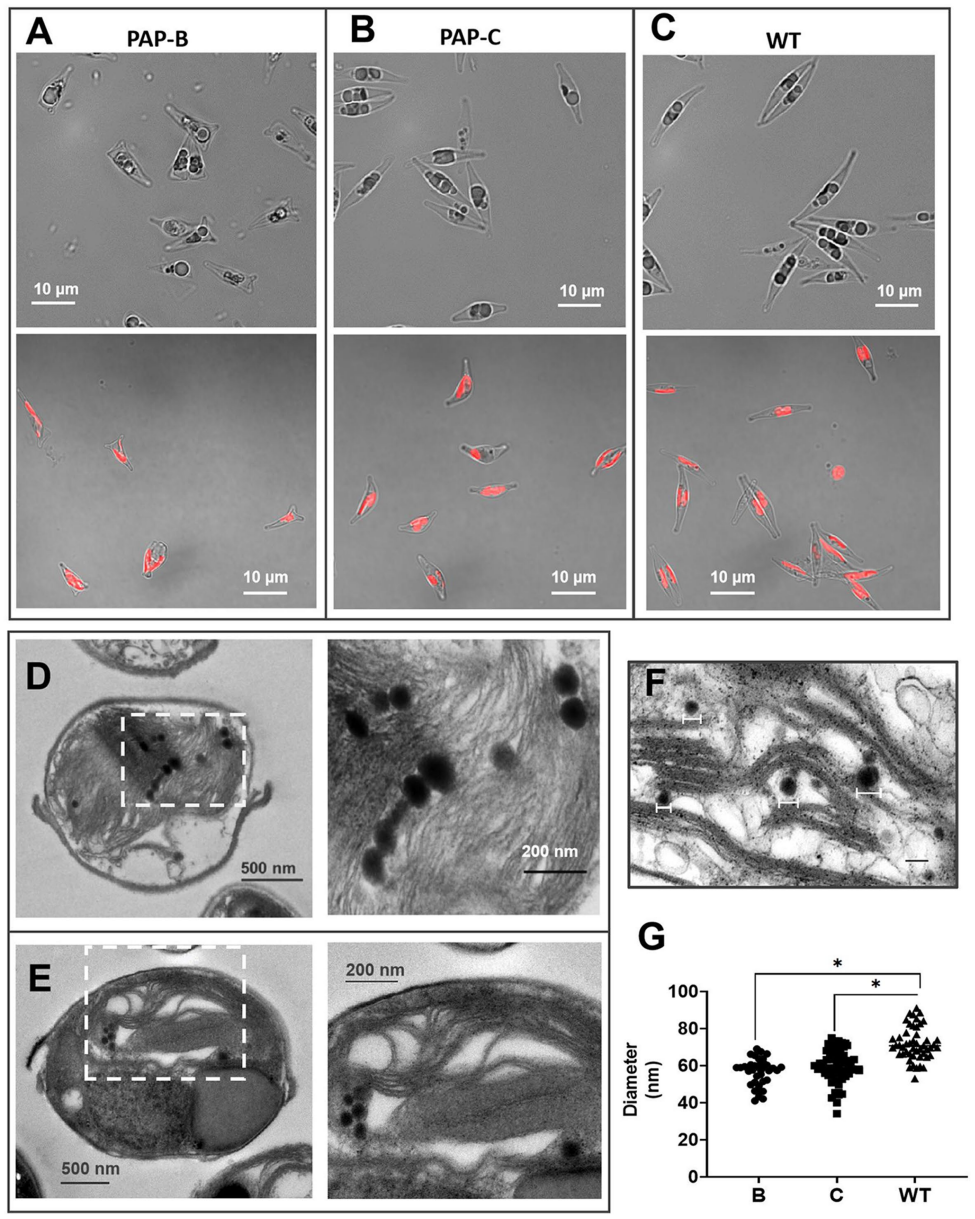
Morphological analysis of the mutants and WT. **(A–C)** Micrographs of PAP-B, PAP-C, and WT. The bottom images present the distribution of spontaneous chlorophyll fluorescence in cells. **(D,E)** Transmission electron microscopy (TEM) images of the WT and mutants. The right image is a partial enlargement of the left image. **(F)** ImageJ was used to calculate the diameters of PGs in the TEM images. **(G)** Statistical analysis of the PG diameters in different cells.

Endogenous *PAP* was overexpressed by electroporation. Gene insertions were achieved by random integration. Among the obtained mutant strains, six strains (named PAP-A ~ F) were selected for subsequent analysis. At the initial stage of cultivation, microscopic observation revealed apparent differences in cell morphology, particularly an increase in transverse diameter and a decrease in the longitudinal diameter between the mutant and WT strains. The mutant strain PAP-B displayed a triangular cell morphology. Statistical analysis was performed in 15 random microscopic fields (six mutants were selected for observation, and the cell density was 6 × 10^7^ cells∙mL^−1^ during observation). The mutants were predominantly larger than the WT in the transverse diameter. In particular, the transverse diameter was more than 10% larger than that of the WT in 92.3% of the mutants. Triangular-shaped cells accounted for 85.5% of the total cells of PAP-B at the initial stage of cultivation (Figures 2A–C). We speculate that overexpression of *PAP* affects the cell wall and cytoskeleton, thereby causing changes in cell morphology. This phenomenon was neither expected nor has it been reported.

Furthermore, PAP-B and PAP-C, which had the most extensive morphological differences among the mutant strains, were selected for TEM observation of PG structures (Figures 2D,E). First, the numbers of PGs did not significantly differ among different strains because of the differences in the choice of cell section. Different from the number of PG structures, the size of PGs was not affected by the choice of cell section. Therefore, we compared the diameter between the mutants WT, observing that the diameters of PGs were smaller in the mutants (Figures 2F,G). This phenomenon supports the existence of a phenotypic association between the predicted functional protein PHATRDRAFT_55153 and the PGs. The expression of this PAP protein might affect the size and structure of PGs. Further validation of the protein function might require immunoelectron microscopy-based approaches for quantitative analysis.

### 3.3. An essential role of the PAP gene in photosystem assembly

**Figure 3:**
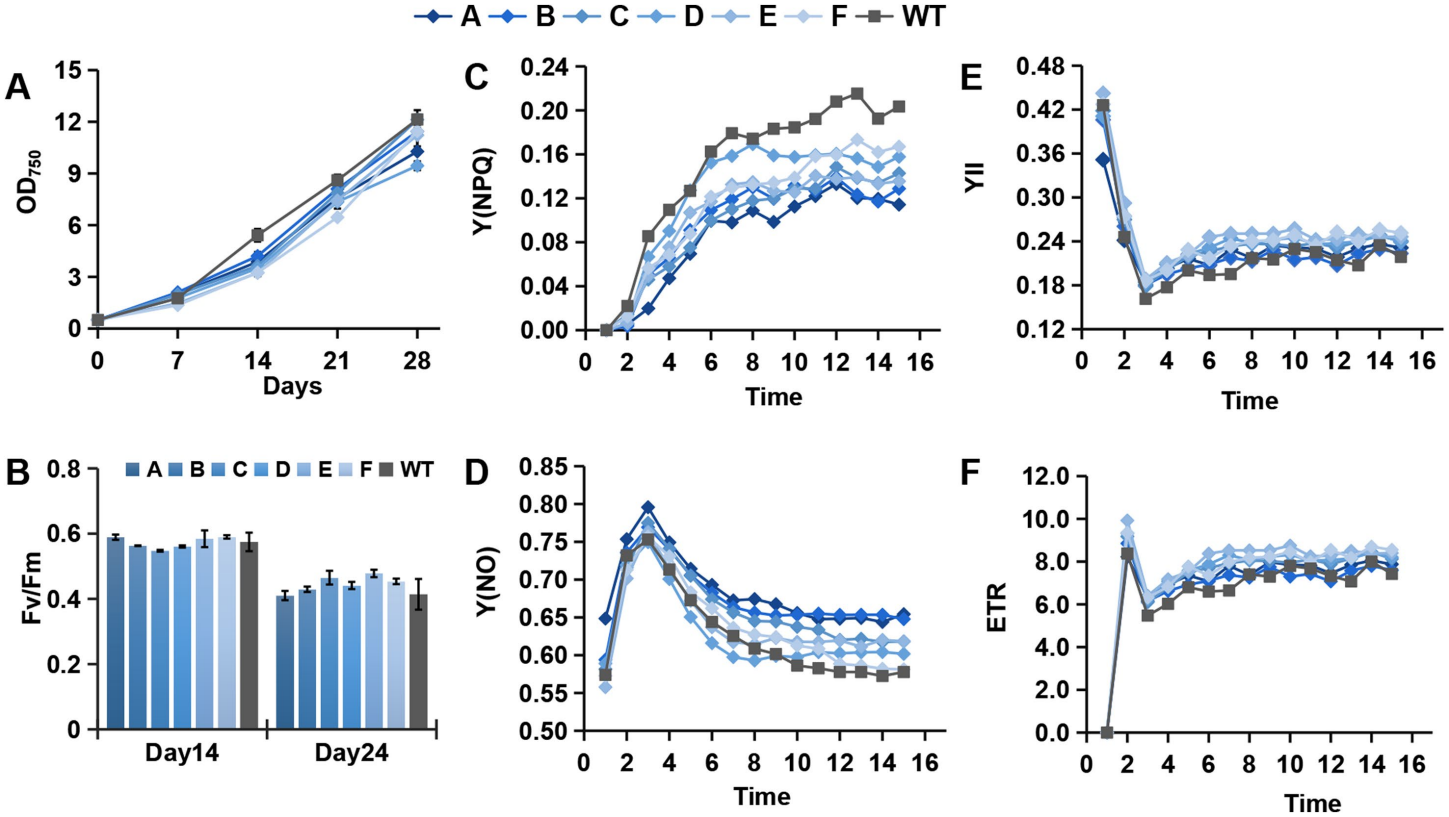
Growth and photosynthetic parameters of different mutants and the WT. **(A–F)** Growth curve, Fv/Fm, Y(NPQ), Y(NO), YII, and ETR of the photosystem.

The mutants and WT were incubated with shaking under the same mixotrophic conditions. Comparing the growth curve by measuring OD_750_, the mutant strains grew slower than the WT. With increasing cell passage, the differences in growth rates almost disappeared. The cell growth curves after three passages (generation time = 20 days) are presented in Figure 3A. The change in growth rates was synchronous with the aforementioned differences in cell morphology. PCR-based analysis of the genome found no loss of the transformed gene fragments. Changes in the cell morphology and growth rate might have resulted from adaptive evolution.

The photosynthetic parameters of the mutants and WT were determined during growth. There was no significant difference in Fv/Fm between the mutants and WT (Figure 3B). However, in the photosynthetic kinetics analysis, NPQ was significantly larger in the WT than in the mutants, whereas Y(NO) was lower in the WT than in the mutants (Figures 3C,D). These results indicate that the WT can more actively shield the photosystem from destruction under high light conditions. The mutants also had a slightly higher photosynthetic electron transport rate (ETR) and Y(II) than the WT (Figures 3E,F). This series of changes in photosynthetic parameters illustrate that the efficiency of the photochemical reaction center was not affected, whereas the changes mainly focused on light-harvesting antenna proteins, especially heat dissipation-associated molecules. NPQ is one of the most rapid mechanisms diatoms possess to dissipate excess energy. Its capacity is mainly defined by the xanthophyll cycle (XC) and light-harvesting complex X (Lhcx) proteins (Blommaert et al., 2020). The diadinoxanthin (Ddx) de-epoxidation is one of the fastest biochemical responses of the thylakoid membrane to environmental factors (Bojko et al., 2019). Previous studies described a carotenoid biosynthesis pathway in PGs, implying that PGs in chloroplasts are the essential structures for carotenoid biosynthesis (Deruere et al., 1994a; Vishnevetsky et al., 1999; Ytterberg et al., 2006; Singh and McNellis, 2011). We speculate that the changes in PGs associated with PAP genes affect the synthesis of intracellular xanthophylls that bind photosynthetic antenna proteins, which will significantly affect the efficiency of NPQ. Therefore, we further analyzed the contents of photosynthetic pigments.

### 3.4. Overexpression of the PAP gene improved fucoxanthin and Chl-*a* levels

**Figure 4:**
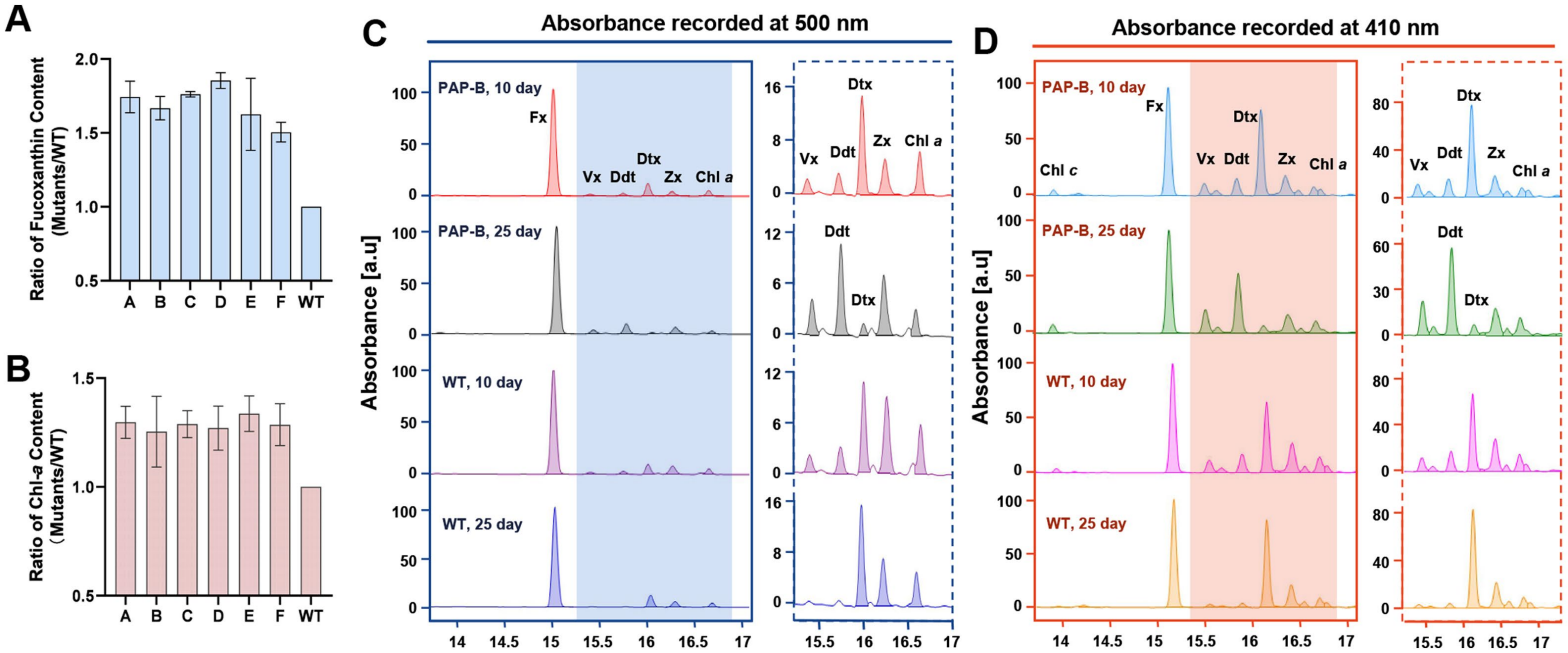
Pigment composition of the mutant PAP-B and WT. **(A,B)** The ratio of fucoxanthin and Chl-a content between the mutant and WT after 14 days. **(C,D)** The pigment compositions of PAP-B and WT were analyzed by HPLC at different culture times, and the absorption peaks at 500 and 410 nm are presented.

In this study, the intracellular content of fucoxanthin during cultivation was analyzed. The results illustrated that the fucoxanthin content was higher in the mutants than in the WT in different cultivation periods. The PAP-D strain exhibited 85.39% higher fucoxanthin content than the WT after 14 days of cultivation (Figure 4A). On day 24 of cultivation, the fucoxanthin content of mutants was on average 33.10% higher than that of the WT. In addition, the intracellular content of Chl-*a* was analyzed. The trend of Chl-*a* content was consistent with that of fucoxanthin. The mutants had an average 28.85% higher Chl-*a* level than the WT after 14 days of culture (Figure 4B). On day 24, this difference decreased to an average of 15.62%. The increase in Chl-*a* content also made the PAP-B cells appear dark green in a short period rather than reflecting the brown color of diatoms. The expression of the predicted PAP protein resulted in enhanced fucoxanthin and chlorophyll levels in the mutants. In *P. tricornutum*, the crystal structure of FCP reveals that the Lhcf4 protein binds seven Chl-*a*, two Chl-*c*, seven fucoxanthin, and probably one Ddx moiety (Wang H. et al., 2019). The assembly of FCPs and pigments into a complex, followed by further assembly with a photochemical reaction center into a super complex, is responsible for converting light energy into chemical energy (Pi et al., 2019; Wang et al., 2020). However, the proportion of this pigment content is not constant, as the xanthophyll content in particular can vary with the growth environment. Significantly, marine algae experience non-periodic fluctuations in their exposure to light because of water mobility. In diatoms, NPQ is associated with the transformation and accumulation of the XC pigments Ddx and diatoxanthin (Dtx) (Büchel, 2020; Kuczynska et al., 2020). Their concentrations determine the magnitude of the NPQ response. Under high light, FCPs increase their XC pigment content and the de-epoxidation ratio (Lepetit et al., 2012), thereby enhancing their capacity to dissipate energy (Gundermann and Büchel, 2012). Enrichment of Dtx in PSII under high light suggests that photosystems are organized to allow xanthophylls to remain in dynamic balance. This means that xanthophylls are not synthesized and decomposed continuously, but require a storage structure near the photosystem. Combined with the change of PG structures, we speculate that *PAP* overexpression leads to the formation of a more high-density isopentene microcompartment, which affects the exchange of xanthophylls on the thylakoid membrane and PGs, thus changing the assembly of the photosystem.

When the pigment composition of the mutant PAP-B and the WT was analyzed by HPLC, a significant difference was observed in the levels of Ddx and Dtx. The Ddx level was significantly higher than that of Dtx in PAP-B at the later stage of culture. In contrast, the Ddx level was much lower than the Dtx level in PAP-B during the early stage and in the WT during different periods. Ddx should be de-epoxidated to Dtx under light on the thylakoid membrane (Figures 4C,D), whereas the mutant strain at the later stage cultured under the same conditions had a high level of Ddx, most likely because Ddx cannot be integrated into thylakoid membranes nearby the light-harvesting antenna proteins and therefore it was not de-epoxidated. Combined with the previous speculation, a significant amount of Ddx might be “trapped” in the PGs without transport into the photosynthetic system complex.

### 3.5. Transcriptome data analysis

The transcriptome data of PAP-B, which featured the most significant change in cell morphology, were compared with those of the WT. We selected the cells cultured on day 14 for transcriptomic analysis because the morphology of microalgae cultured to this stage was relatively stable and the difference in the pigment content reached its maximum.

**Figure 5:**
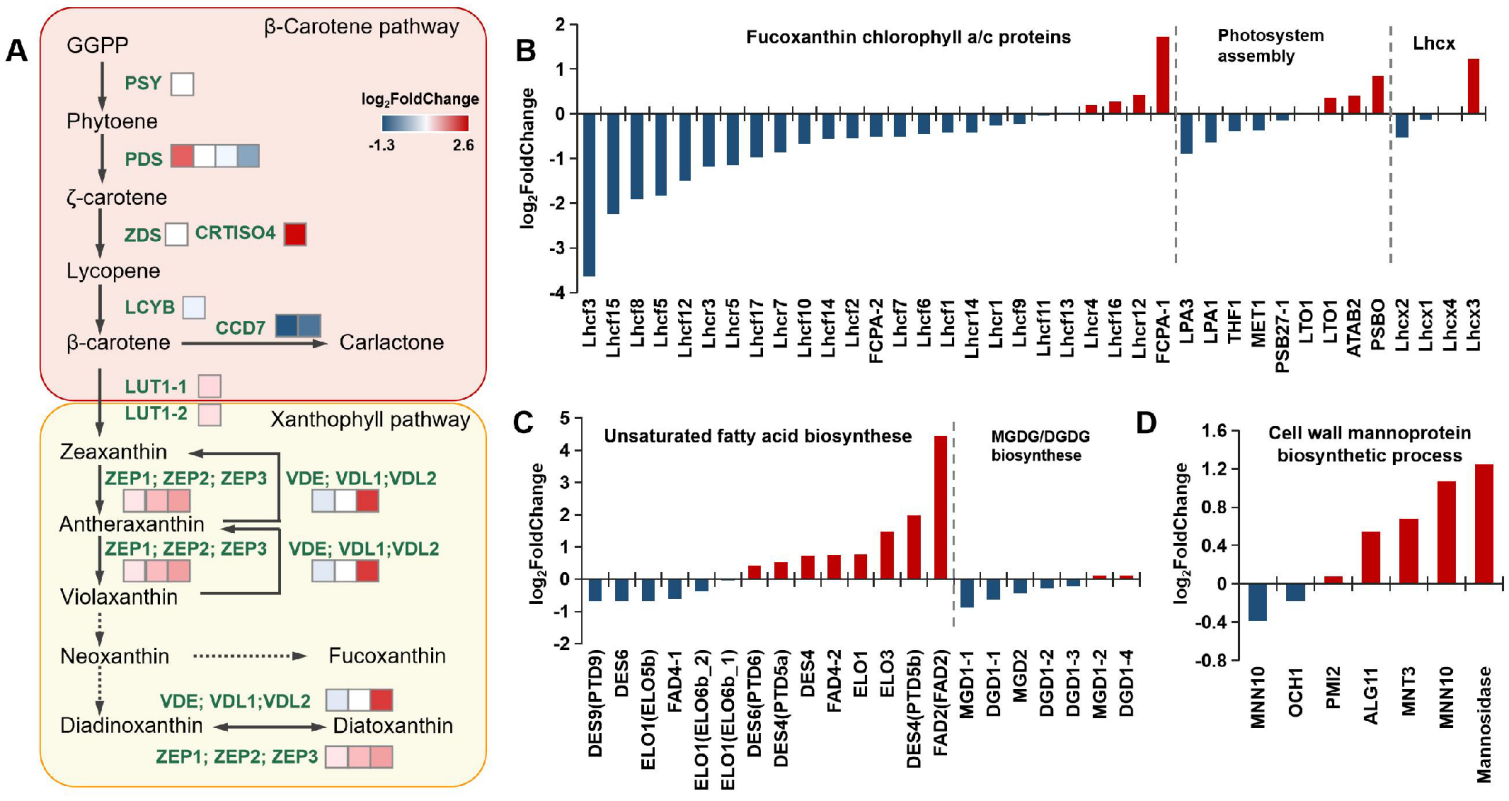
Transcriptomic analysis of different metabolic pathways. **(A)** Expression of genes in the carotenoid synthesis pathway. **(B)** Expression of genes in the FCP complex, photosystem assembly, and the *Lhcx* genes. **(C)** Expression of genes in the polyunsaturated fatty acid synthesis pathway and MGDG/DGDG synthesis pathway. **(D)** The mannose metabolism genes are related to cell wall synthesis. All abbreviations and data are provided in Supplementary Data 2.

First, we focused on the expression of all annotation genes in the carotenoid synthesis pathway. The most strongly upregulated gene was *CRTISO4* (log_2_FC = 2.57), which encodes carotenoid isomerase. Recently, this gene was also proven to participate in the cis-trans isomerization of phytoene, a key gene for lycopene formation (Sun et al., 2022). In addition, most of the enzymes in the fucoxanthin synthesis pathway were upregulated (Figure 5A), which was consistent with increased fucoxanthin content. In addition, XC-related enzymes were also significantly upregulated. The transcriptome data also revealed significant upregulation of the violaxanthin de-epoxidase-like gene (PHATRDRAFT_46155). The upregulation of this enzyme is usually accompanied by stress, which is required to initiate the Ddx cycle on the thylakoid membrane (Goss and Latowski, 2020).

The most strongly downregulated gene was carotenoid cleavage dioxygenase, which can catalyze the oxygenolytic fission of alkene bonds in carotenoids to generate apocarotenoid products (Zhou et al., 2019). The downregulation of degradation pathways in the mutant strains was accompanied by the accumulation of carotenoids.

Transcriptomic analysis of the chlorophyll synthesis and degradation pathways in mutants revealed that most of the key genes of the chlorophyll synthesis pathway were upregulated. In contrast, most of the genes of the chlorophyll degradation pathway were downregulated. In particular, the pheophytin pheophorbide hydrolase (pheophytinase, *PPH*) gene was significantly downregulated. PPH is a critical enzyme in Chl degradation. Mutagenesis or overexpression of *PPH* can lead to a stay-green or premature senescence phenotype in *Arabidopsis* and rice (Schelbert et al., 2009; Wang W. et al., 2019). Previous studies identified PPH in isolated PGs, indicating that PPH is likely a *bona fide* PG protein (Lundquist et al., 2012b).

Further analysis of the transcriptome data demonstrated that most antenna proteins were downregulated (Figure 5B). Previous studies on the FCP crystal structure in *P. tricornutum* revealed that functional PSII–FCPII monomers include one PSII core, two FCP tetramers, and three FCP monomers (FCP-D/E/F). One of the FCP tetramers is directly associated with the core at the CP47 side and designated strongly associated tetramer [S-tetramer (ST)], whereas the other one is associated with the PSII core indirectly at the CP43 side through two FCP monomers, FCP-D and FCP-E, and hence designated moderately associated tetramer [M-tetramer (MT)] (Wang et al., 2020). The transcriptome results for FCP complexes illustrated that only a few antenna proteins were upregulated, and most FCP antenna proteins were downregulated. This might lead to the inhibition of antenna protein assembly.

PG structures enable the synthesis and storage of intracellular carotenoid substances. The protein structural properties of PAP might increase the ability of PGs to store and bind carotenoid substances, thus reducing carotenoid transport into the photosynthetic system. This is responsible for the reduced synthesis of the corresponding antenna proteins during the assembly of the photosynthetic system.

Lundquist speculated that PGs are essentially microdomains within the thylakoid membrane that potentially serve as platforms to recruit proteins and metabolites to facilitate metabolic channel activity or signal transduction (Lundquist et al., 2013). We further suggest that PGs act as a pool, and alterations in PAP protein expression might affect pigment metabolism in the thylakoid membrane, resulting in unexpected modifications in photosynthetic regulation.

Due to the NPQ difference between mutants and WT, we also detected the expression of *Lhcx* genes in the photosynthetic system. Diatoms possess an impressive capacity of NPQ, provided by the xanthophyll diatoxanthin and Lhcx proteins, and there are four *Lhcx* genes in *P. tricornutum* (Buck et al., 2019). The results showed that *Lhcx2* in the mutant strain was significantly downregulated, while the expression of *Lhcx3* was upregulated considerably (Figure 5B). However, the expression level of *Lhcx3* in different strains is very low (Supplementary Data 2). In this case, the difference in NPQ between the mutant and WT may be more influenced by *Lhcx2* and the Ddx/Dtx ratio.

In addition to carotenoid and photosystem metabolism, we performed transcriptomic analysis of lipid metabolism in the mutants (Figure 5C). The chloroplast thylakoid membrane is mainly composed of lipids and different protein–pigment complexes. The hydrophobic region inside the membrane bilayer interacts with membrane proteins to ensure that the light energy absorbed by the pigment causes the production and transmission of photosynthetic electrons and the proper progression of photophosphorylation. The major lipid type in the thylakoid membranes of photosynthetic organisms is polar glyceride, which includes three glycolipids [monogalactosyl diacylglycerol (MGDG), digalactosyl diacylglycerol (DGDG), and sulfoquinovosyl diacylglycerol (SQDG)] and phospholipid (phosphatidyl glycerol). Among these glycerides, MGDG and DGDG, which contain high proportions of PUFAs, accounted for approximately 50 and 30% of the total lipid content, respectively (Goss and Latowski, 2020). A high proportion of PUFAs is believed to be related to the high fluidity of the membrane, and it is an essential feature for efficient photochemical reactions on thylakoid membranes. In addition, 20 DGDG, 42 MGDG, 16 SQDG, and 30 phosphatidylglycerol molecules are found in a PSII–FCPII dimer. These lipids are mostly distributed in the interfaces between subunits, suggesting their roles in mediating subunit interactions (Wang et al., 2020). In the transcriptomic analysis, most genes of the PUFA synthesis pathway were upregulated, especially delta (12)-fatty-acid desaturase 2. However, the expression of enzymes synthesizing MGDGs and DGDGs did not significantly differ (Figure 5C). We analyzed the fatty acid composition of the mutants and WT using GC. The results indicated that the proportion of EPA present in each mutant was significantly higher than that in the WT. This was directly linked to the significant upregulation of fatty acid desaturases identified from the transcriptomic analysis (see Table 1).

**Table 1:** Fatty acid composition of the WT and transformants.

Strains	EPA (%)	SFA^a^ (%)	MUFA^b^ (%)	PUFA^c^ (%)
A	19.82 ± 0.02	31.37 ± 0.22	30.52 ± 0.49	38.1 ± 0.71
B	22.12 ± 0.15	31.86 ± 0.13	29.06 ± 0.46	39.07 ± 0.33
C	21.48 ± 0.13	38.26 ± 0.07	26.12 ± 0.45	35.60 ± 0.52
D	20.21 ± 0.25	35.63 ± 0.39	28.13 ± 0.21	36.23 ± 0.59
E	24.5 ± 0.13	30.64 ± 0.07	24.15 ± 0.05	45.20 ± 0.02
F	18.29 ± 0.13	34.40 ± 0.33	31.96 ± 0.18	33.63 ± 0.49
WT	15.38 ± 0.54	41.19 ± 1.01	31.27 ± 0.12	27.52 ± 1.13

a indicate the saturated fatty acids, monounsaturated fatty acids, and polyunsaturated fatty acids, respectively.

b indicate the saturated fatty acids, monounsaturated fatty acids, and polyunsaturated fatty acids, respectively.

c indicate the saturated fatty acids, monounsaturated fatty acids, and polyunsaturated fatty acids, respectively.

By isolating lipids based on their polarity, we purified the neutral lipids, glycolipids, and phospholipids for GC analysis. The results revealed that PUFAs such as EPA levels of the mutants were significantly higher than that of the WT in the fatty acid composition of glycolipids. In contrast, no significant differences were found in neutral lipids and phospholipids (Supplementary Figure 1; Supplementary Table 1). PUFAs such as EPA mainly accumulate in membrane lipids. This indicates the increase of membrane fluidity during the assembly of the photosynthetic system. Consequently, the interactions between the thylakoid membranes and the PS–FCP subunits would be induced.

Finally, we focused on cell wall-related metabolism, as significant differences in cell morphology were observed upon phenotype comparisons. The cell wall of *P. tricornutum* has low silica content, differing from other diatoms, which are mainly composed of organic molecules, notably sulfated glucuronomannan (Le Costaouëc et al., 2017). The polysaccharide backbone consists of a mannan chain decorated with sulfate ester. It was proposed that the branching consists of mannose and glucuronic acid based on structural analyses of fragments obtained by mild acid hydrolysis. We mainly analyzed mannose metabolism genes involved in the cell wall synthesis pathway. The results indicated that the related genes (alpha-mannosidase, alpha-1,6-mannosyltransferase, alpha-1,2-mannosyltransferase) were upregulated (Figure 5D). Because the cell wall synthesis pathway in *P. tricornutum* is unclear, there was no differential comparison of other annotated genes related to cell wall synthesis.

Previous studies on different morphological cells of *P. tricornutum* illustrated that the cell wall of *P. tricornutum* exhibits high plasticity compared to those of other diatoms, and it can display three morphotypes: fusiform, oval, and triradiate. In comparison, the cellular morphology of our mutant strains appeared to be intermediate between fusiform and oval. *P. tricornutum* cells will undergo different physiological changes upon exposure to environmental stress. In particular, comparative transcriptomic analysis based on EST indicated that the oval morphotype features the upregulation of genes encoding proteins involved in hyposalinity and/or cold stress responses. Meanwhile, the plasticity of the cell wall is also sensitive to different stress conditions (Vartanian et al., 2009; Ovide et al., 2018; Galas et al., 2021).

## 4. Discussion

Chloroplast PGs are dynamic monolayer membrane structures containing special metabolites and proteins. They store secondary metabolites such as pigments and play an active role in the developmental transition and environmental adaptation, making them microcompartments with integrated functions. A comprehensive functional model of PGs was constructed by co-expression analysis using PG proteins, and the co-expression network was involved in four specific functions of PGs: (1) senescence, (2) plastid biogenesis, (3) isoprenoid lipid metabolism, and (4) redox/photosynthetic regulation (Lundquist et al., 2012b).

**Figure 6:**
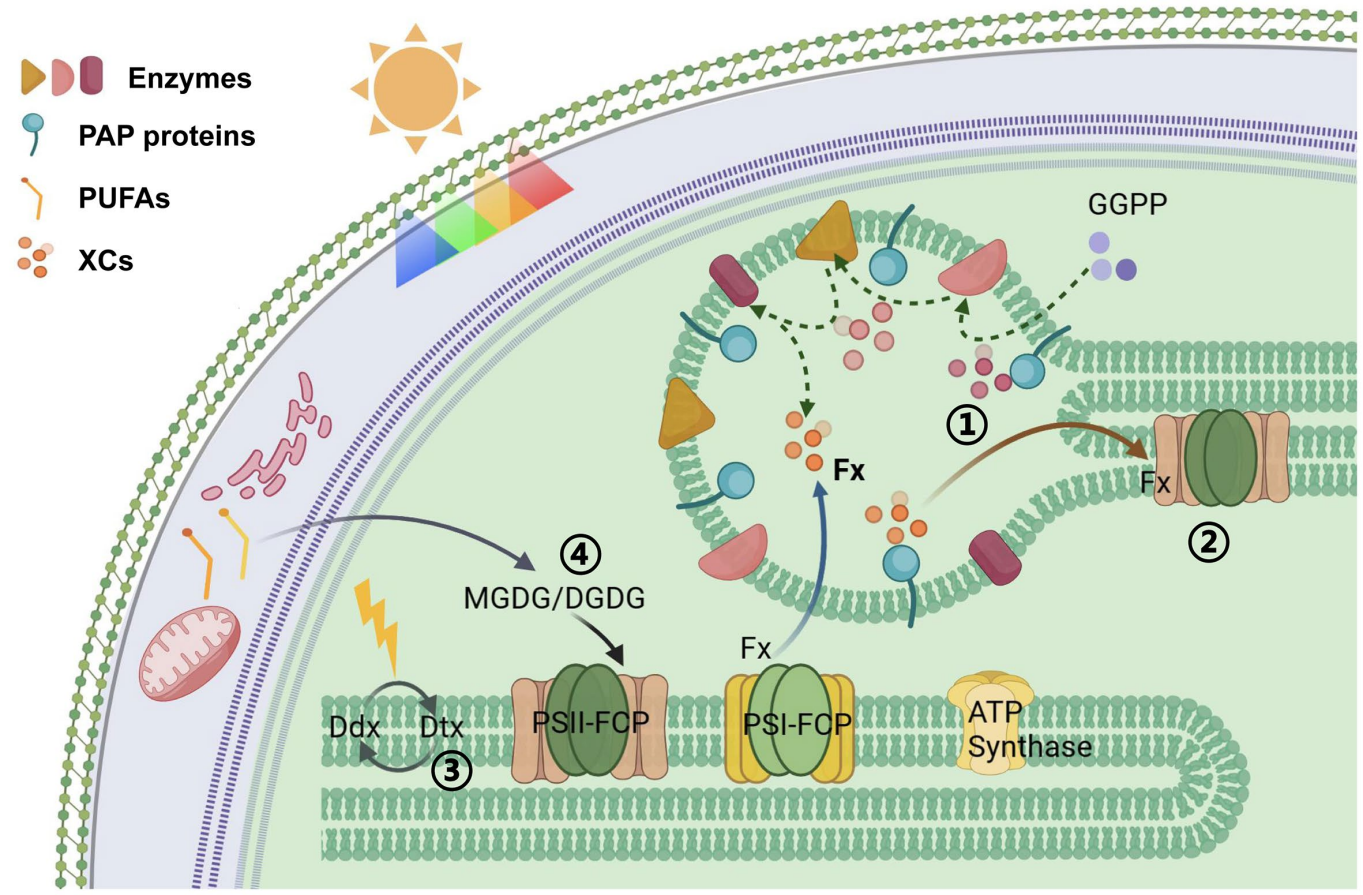
Schematic overview of proposed organization: Overexpression of the *PAP* gene (1) led to the accumulation of xanthophylls in PGs, (2) affected the assembly of photosynthetic system antenna proteins, (3) reduced the efficiency of XC in thylakoids, (4) increased the degree of unsaturation of MGDG and DGDG in thylakoid membranes, thereby enhancing membrane fluidity.

This study obtained a series of mutants by overexpressing a predicted PAP gene. We unexpectedly found that the cell morphology of the mutants all shifted toward an oval morphotype. In phenotype detection, the unified changes of the mutants typically included increases in the levels of fucoxanthin and PUFAs, especially EPA, and a decrease in NPQ activity. PGs are located on the thylakoid membrane and extended out by the thylakoid membrane. Therefore, the changes in PGs and cells were caused by *PAP* overexpression, which we believe is closely related to the assembly and function of the photosystem (Figure 6).

First, the diameter of PGs became smaller after *PAP* overexpression. Combined with the previous model, changes in PG inclusions affect PG size (Lundquist et al., 2013). The nonpolar components are buried inside the PGs and covered by polar lipids and proteins on the surface of PGs. The protein structure of PAP renders it amphipathic. Its N-terminal ɑ-helix can bind to the polar lipid of the monolayer, and the β-sheets of the tail constitute a barrel structure that could bind relatively hydrophobic substances. The ratio of amphiphilic-to-hydrophobic metabolites in PGs is reflected in their surface area/volume ratio and thus the diameter of the approximately spherical PGs. When the overexpressed PAP protein is located on the PG surface of *P. tricornutum*, more surface sites are occupied by the protein, which might more specifically bind to isoprenoids inside the PGs. Consequently, isoprenoids are more strongly accumulated in the interior of PGs than other metabolites, and this selectivity might lead to a decrease in the spherical volume. In fact, we suggest that the structural properties of this PAP protein determine the preference of PGs for the encapsulated contents.

In diatoms, a Ddx–Dtx cycle exists in the FCP antennae, and it quenches excess energy under strong light conditions. In our study, the photosynthetic electron transport efficiency and photosynthetic efficiency of the mutants did not show an obvious decrease, whereas NPQ was decreased. We speculated that increases in PAP protein levels on PGs and their binding capacity for isopentenyls affected the storage and transport efficiency of xanthophylls. Consequently, the exchange efficiency of substances between PGs and photosynthetic systems and the dynamic equilibrium of xanthophylls in these systems were altered. Regulating the number of PGs and the expression of PAP proteins appears to play a role in pigment metabolism in the photosynthetic system. We suggest that PGs act as buffering pools for carotenoids in plant cells, making them essential for regulating the photosynthetic efficiency of diatoms.

To use *P. tricornutum* as an industrial microorganism for production, unlike the natural environment in which microalgae need to adapt to constant changes in light and temperature; in the production process, especially in mixotrophic fermentation, the environment of the algal cells is relatively stable regarding light and temperature. Under this circumstance, engineering algal species to obtain strains that efficiently accumulate fucoxanthin at the expense of reduced redundant NPQ capacity can be used as a strategy for algal species modification in the future.

EPA is another high-value product of *P. tricornutum*. The content of this PUFA was also elevated in the mutant strains. We also believe that there is a close relationship between the accumulation of EPA and the assembly of the photosynthetic system of the cell. In the membrane lipids of thylakoids of diatoms, MGDG acts as a solvent for XC pigments (Latowski et al., 2004). EPA preferentially binds to the sn-1 position of the glycerol backbone in MGDGs. DGDGs exhibit a fatty acid component comparable to that of MGDGs (Dodson et al., 2014). Similar to MGDG, EPA generally occupies the sn-1 position of DGDGs. In thylakoid membranes, MGDG appears to play a key role in providing membrane fluidity, which is essential for the efficient diffusion of XC pigments (Goss and Latowski, 2020).

Finally, in terms of changes in cell morphology, we suggest that both the synthesis and metabolism of the cell wall affect cell morphology. These processes are also associated with alterations in the cellular content and cytoskeleton. Many studies have examined the cell wall formation of diatoms and its alteration with changes in the spectra (Vartanian et al., 2009). The morphology of *P. tricornutum* is easily comparable to that of a convex lens. The cell morphology and its contents cause light refraction, which is consistent with the distribution of the photoreactive centers of chloroplasts and the advantages of the FCP complex in absorbing spectra of different wavelengths. In our study, the morphological changes of the mutants might increase the refractive index of the “convex lens,” thus having a great refractive effect on the spectrum. As an explanation, we tend to attribute this phenomenon to the lack of lutein-like substances dependent on the assembly of the FCP complex, resulting in the dependence on specific spectra. In addition, we found that transitions between multiple morphologies of *P. tricornutum* are probably attributable to the spectral dependence of the photosystem.

PGs are structures of subcellular organelles. The single-layered membranous structure formed on the thylakoid gives it close contact with the photosynthetic system for the exchange of substances. Our study preliminarily revealed the role of PGs as a pool for synthesizing and storing xanthophylls in photosynthetic systems in a primitive marine diatom. This finding inspires the utilization of PGs in metabolic engineering. This subcellular organelle structure can be modified and engineered as a functional block, thus providing a probable target site for synthetic biology.

## Data availability statement

The datasets presented in this study can be found in online repositories. The names of the repository/repositories and accession number(s) can be found in the article/Supplementary material.

## Author contributions

E-YJ, YF, and W-YX accomplished the gene isolation, transformation, positive transformants screening, and carotenoid analysis. G-RH and F-LL supervised the experiments and provided guidance. W-YX and N-VP contributed to the fatty acid analysis. YF and E-YJ designed the experiment, analyzed the data, and drafted the manuscript. All authors contributed to the article and approved the submitted version.

## Funding

This work was supported by the National Key Research and Development Program of China (2021YFA0909703; 2019YFD0901904), The National Natural Science Foundation of China (31973007), Shandong Energy Institute (SEI I202136).

## Conflict of interest

The authors declare that the research was conducted in the absence of any commercial or financial relationships that could be construed as a potential conflict of interest.

## Publisher’s note

All claims expressed in this article are solely those of the authors and do not necessarily represent those of their affiliated organizations, or those of the publisher, the editors and the reviewers. Any product that may be evaluated in this article, or claim that may be made by its manufacturer, is not guaranteed or endorsed by the publisher.
