# Supplementary material for: Overexpression of plastid lipid-associated protein in marine diatom enhances the xanthophyll synthesis and storage
Source: Front Microbiol. 2023 Apr 21;14:1143017. doi: 10.3389/fmicb.2023.1143017 (PMC10160619; doi:10.3389/fmicb.2023.1143017)
Supplement: Supplementary file 1 [file Data_Sheet_1.docx]

Supplementary Material

Overexpression of plastid-lipid-associated protein in marine diatom enhances the xanthophyll synthesis and storage

Er-Ying Jiang^1,4^, Yong Fan^1,*^, Nghi-Van Phung^1^, Wan-Yue Xia^1^, Guang-Rong Hu^1^, Fu-Li Li^1,2,3,*^

* **Correspondence:**

Fu-Li Li: [lifl@qibebt.ac.cn;](mailto:lifl@qibebt.ac.cn;) Yong Fan: [fanyong@qibebt.ac.cn](mailto:fanyong@qibebt.ac.cn)

# Supplementary Data 1

Sequences used for evolutionary tree analysis. Sheet-1 is the genes list; Sheet-2 is the protein sequences.

# Supplementary Data 2

Analysis of transcriptome data on different metabolic pathways. All abbreviations and data in figure 5 are provided in this file.

**Supplementary Figure 1.** EPA content comparison of total lipids and different lipid fractions between WT and mutants. The figure shows the average of three independent replicates ± SE


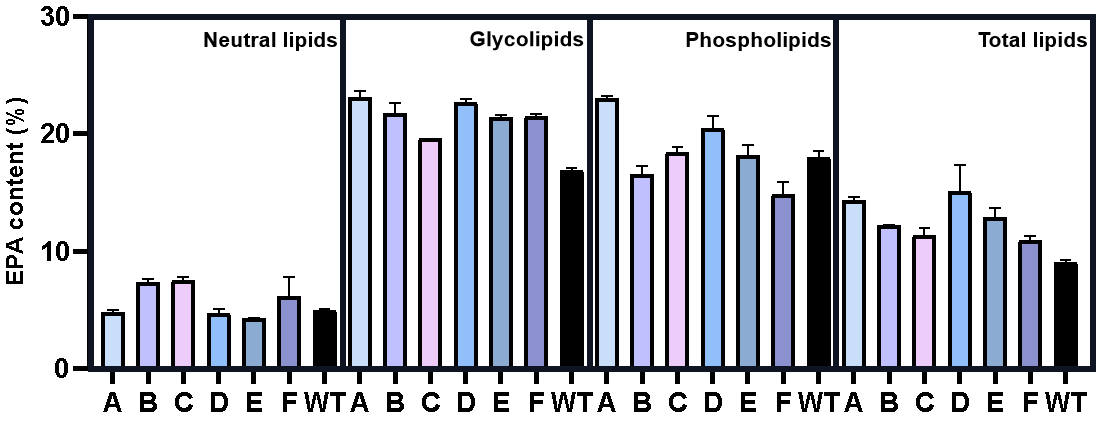


**Supplementary Table 1.** Fatty acid composition of total lipids and different lipid fractions between WT and mutants.

|  |  | **A** | **B** | **C** | **D** | **E** | **F** | **WT** |
| --- | --- | --- | --- | --- | --- | --- | --- | --- |
| **EPA**  **(%)** | **Total lipids** | 14.18±0.19 | 12.26±0.01 | 11.04±0.38 | 16.73±1.59 | 13.46±0.52 | 10.62±0.29 | 9.23±0.14 |
|  | **Neutral lipids** | 4.97±0.15 | 7.56±0.20 | 7.71±0.21 | 4.52±0.25 | 4.28±0.02 | 7.3±1.15 | 4.94±0.05 |
|  | **Glycolipids** | 22.76±0.38 | 21.19±0.60 | 19.63±0.00 | 22.95±0.21 | 21.32±0.14 | 21.63±0.11 | 16.85±0.11 |
|  | **Phospholipids** | 22.88±0.17 | 17.08±0.51 | 18.13±0.32 | 19.75±0.74 | 18.8±0.64 | 15.6±0.70 | 17.62±0.40 |
| **SFA**  **(%)** | **Total lipids** | 44.42±0.07 | 46.3±0.04 | 45.97±0.2 | 52.05±5.53 | 41±0.15 | 42.83±0.03 | 45.07±0.01 |
|  | **Neutral lipids** | 56.93±0.05 | 52.94±0.46 | 51.62±0.17 | 59.18±0.32 | 59.22±0.54 | 32.89±9.59 | 52.89±0.03 |
|  | **Glycolipids** | 44.13±0.12 | 47.2±0.20 | 50.05±0.59 | 44.55±0.57 | 46.3±0.59 | 41.03±0.20 | 53.22±0.36 |
|  | **Phospholipids** | 47.98±0.62 | 55.66±1.65 | 54.45±0.17 | 51.37±0.30 | 50.64±0.34 | 54.24±0.30 | 53.54±2.00 |
| **MUFA**  **(%)** | **Total lipids** | 28.14±0.22 | 30.62±0.04 | 33.4±0.37 | 25.84±1.32 | 29.04±0.47 | 34.29±0.73 | 36.52±0.17 |
|  | **Neutral lipids** | 28.32±0.08 | 30.95±0.20 | 33.24±0.24 | 27.71±0.11 | 28.49±0.16 | 50.13±7.29 | 36±0.00 |
|  | **Glycolipids** | 20±0.28 | 21.69±0.96 | 21.98±0.26 | 21.64±0.05 | 21.28±0.15 | 25.88±0.20 | 19.99±0.21 |
|  | **Phospholipids** | 25.43±0.09 | 27.26±2.33 | 25.15±0.59 | 24.35±0.62 | 25.68±1.23 | 26.5±0.03 | 25.46±2.09 |
| **PUFA**  **(%)** | **Total lipids** | 27.45±0.29 | 23.08±0.08 | 20.63±0.57 | 22.11±4.21 | 29.96±0.32 | 22.88±0.71 | 18.41±0.15 |
|  | **Neutral lipids** | 14.75±0.13 | 16.11±0.65 | 15.14±0.08 | 13.1±0.44 | 12.29±0.38 | 16.97±2.30 | 11.11±0.03 |
|  | **Glycolipids** | 35.87±0.40 | 31.11±0.77 | 27.96±0.33 | 33.81±0.63 | 32.42±0.74 | 33.09±0.00 | 26.79±0.15 |
|  | **Phospholipids** | 26.59±0.53 | 17.08±0.67 | 20.4±0.42 | 24.28±0.32 | 23.67±1.58 | 19.25±0.26 | 21±0.10 |

The table shows the average of three independent replicates ± SE.
